# Personals

**Published:** 1914-02

**Authors:** 


### PERSONALS

***Announcement of removal, travel, and other matters of interest are requested. Please report errors in the listing of any physician in the State and other directories that we may co-operate with the State Society in securing a correct list.***

Dr. C. A. Vander Beek of Rochester has moved from 44 Gibbs street to 408 Park avenue.

Dr. Edward W. Roos of Corning visited his former home in Buffalo during the holidays.

Dr. Eva G. Fowler of Buffalo is spending several months in Philadelphia, studying diseases of the eye at the Polyclinic.

Dr. John F. Crowley of Batavia has been appointed Physician for the Town Poor at a salary of $450. (Note: Why not pay the City Physicians of Buffalo salaries commensurate with their work and graded to the same level as other employees?)

---

Dr. M. S. Coxe has been appointed Health Officer of Dunkirk.

---

Dr. C. Pearley Lape has removed his residence and office to 979 Lafayette avenue. He is devoting special attention to Anaesthesia and Roentgenography.

---

Dr. Arthur H. Brown gave a dinner to the medical men of Auburn and Cayuga counties on December 23 in commemoration of his thirty years' practice in this city. A loving cup was presented to Dr. Brown by the members present, the presentation speech being made by Dr. J. D. Tripp of Auburn. Dr. O'Neill acted as toastmaster and speeches were made by Drs. W. H. Coe, M. P. Conway, J. P. Creveliry, J. D. Tripp, T. F. Laurie and Dr. A. H. Brown. The dinner was attended by about seventy-five physicians.

